# Patient and Health Care Professional Perspectives About Referral, Self-Reported Use, and Perceived Importance of Digital Mental Health App Attributes in a Diverse Integrated Health System: Cross-Sectional Survey Study

**DOI:** 10.2196/59831

**Published:** 2024-11-15

**Authors:** Michael J Miller, Lindsay G Eberhart, Jennifer L Heliste, Bhaskara R Tripuraneni

**Affiliations:** 1 Mid-Atlantic Permanente Research Institute (MAPRI) Washington, DC United States; 2 Mid-Atlantic Permanente Medical Group Washington, DC United States

**Keywords:** digital mental health applications, DMHA, mobile health, mHealth, mobile phone, smartphone, user experience, engagement, implementation, Kaiser Permanente

## Abstract

**Background:**

Digital mental health applications (DMHAs) are emerging, novel solutions to address gaps in behavioral health care. Accordingly, Kaiser Permanente Mid-Atlantic States (KPMAS) integrated referrals for 6 unique DMHAs into clinical care in 2019.

**Objective:**

This study investigated patient and health care professional (HCP) experiences with DMHA referral; DMHA use; and perceived importance of engagement, functionality, design, and information attributes in real-world practice.

**Methods:**

Separate cross-sectional surveys were developed and tested for patients and HCPs. Surveys were administered to KPMAS participants through REDCap (Research Electronic Data Capture), and completed between March 2022 and June 2022. Samples included randomly selected patients who were previously referred to at least 1 DMHA between April 2021 and December 2021 and behavioral health and primary care providers who referred DMHAs between December 2019 and December 2021.

**Results:**

Of the 119 patients e-mailed a survey link, 58 (48.7%) completed the survey and 44 (37%) confirmed receiving a DMHA referral. The mean age of the sample was 42.21 (SD 14.08) years (29/44, 66%); 73% (32/44) of the respondents were female, 73% (32/44) of the respondents had at least a 4-year college degree, 41% (18/44) of the respondents were Black or African American, and 39% (17/44) of the respondents were White. Moreover, 27% (12/44) of the respondents screened positive for anxiety symptoms, and 23% (10/44) of the respondents screened positive for depression. Overall, 61% (27/44) of the respondents reported DMHA use for ≤6 months since referral, 36% (16/44) reported use within the past 30 days, and 43% (19/44) of the respondents reported that DMHAs were very or extremely helpful for improving mental and emotional health. The most important patient-reported DMHA attributes by domain were being fun and interesting to use (engagement); ease in learning how to use (functionality); visual appeal (design); and having well-written, goal- and topic-relevant content (information). Of the 60 sampled HCPs, 12 (20%) completed the survey. Mean HCP respondent age was 46 (SD 7.75) years, and 92% (11/12) of the respondents were female. Mean number of years since completing training was 14.3 (SD 9.94) years (10/12, 83%). Of the 12 HCPs, 7 (58%) were physicians and 5 (42%) were nonphysicians. The most important HCP-reported DMHA attributes by domain were personalized settings and content (engagement); ease in learning how to use (functionality); arrangement and size of screen content (design); and having well-written, goal- and topic-relevant content (information). HCPs described “typical patients” referred to DMHAs based on perceived need, technical capability, and common medical conditions, and they provided guidance for successful use.

**Conclusions:**

Individual patient needs and preferences should match the most appropriate DMHA. With many DMHA choices, decision support systems are essential to assist patients and HCPs with selecting appropriate DMHAs to optimize uptake and sustained use.

## Introduction

### Background

Anxiety and depressive disorders are common mental health conditions in the United States that cause functional impairment and represent a leading cause of disability [1,2]. Although evidence-based psychotherapy and pharmacotherapy for these conditions exist, the combined shortage of trained mental health clinicians and high demand for mental and behavioral health services have limited access to evidence-based care [3,4]. Thus, there is an urgent need for efficient and effective treatments that are innovative, scalable, and sustainable to address the growing public health dilemma of access to evidence-based behavioral health care services.

Digital mental health applications (DMHAs) are an emerging, novel solution to address this care gap. Many DMHAs apply evidence-based principles and have accumulated empirical support primarily in controlled settings [5-7]. These DMHAs have demonstrated efficacy to reduce mental health symptoms (eg, anxiety and depression), support emotional well-being (eg, stress reduction), and improve resiliency. A recent meta-analysis of 36 randomized clinical trials comparing stand-alone DMHA interventions to non-DMHA control groups found modest therapeutic effect sizes for anxiety (Hedges *g*=0.31) and depression (Hedges *g*=0.35). Key moderators included the duration of the intervention, the presence of symptoms at the start of the study, and the types of outcome measures used [7].

While DMHAs are not a substitute for in-person care, they can be broadly disseminated to at-risk individuals to address subclinical mental health problems before clinical intervention. A stepped-care approach could reduce the demand for the number of patients requiring in-person care for mental health conditions, which would increase the accessibility and availability of in-person health care professionals (HCPs) within health care systems for persons with more immediate or serious need. Beyond this, DMHAs can serve in an adjunctive capacity to in-person evidence-based care within a new and emerging mental health care paradigm.

### Digital Mental Health Integration Into Clinical Care

DMHAs hold considerable potential in complementing accessibility to traditional mental health services. Large health care systems such as Kaiser Permanente (KP) have integrated the use of DMHAs into the management of mental health conditions [8]. Beginning in 2017, KP developed a framework for implementation of DMHAs. As deployed, the DMHAs studied in this research were selected and intended to be used for self-care as part of a comprehensive ecosystem of services for improving emotional health and well-being. Using a human-centered design approach, KP assessed both clinician and patient insights to design a digital health ecosystem [8]. Since then, KP has engaged in ongoing feasibility assessment, implementation, and expansion across its regions and has previously been reported in the literature [8]. Current understanding suggests a need to further explore and refine strategies for implementation and to identify ways to best align candidate patients with the appropriate treatment for a more unified referral and patient experience.

In brief, Kaiser Permanente Mid-Atlantic States (KPMAS) is a large, diverse integrated health care system that provides comprehensive health care services to >800,000 actively insured members in Maryland, the District of Columbia, and Northern Virginia. Health care encounters across the entire care continuum are documented in an electronic health record for all members. All patients in this study were referred to at least 1 of 6 approved DMHAs, recommended by the previously described implementation framework [8], during an in-person or telehealth encounter in outpatient or ambulatory care settings by either a primary care physician, specialty care physician, or a behavioral HCP (physician or nonphysician). HCP workflow was designed to facilitate DMHA referrals using a standardized order set in the electronic health record. Continuing education, training, and individual support where necessary were offered to HCPs at various times during the implementation period. An internal website for HCPs to guide referral of the DMHAs was made available within the workflow. During implementation, behavioral health providers were authorized to refer patients for all 6 DMHAs with no registration cost. However, for primary and specialty care providers, referrals were limited to only 2 DMHAs.

After the initial rollout in December 2019, the KPMAS region observed steep increases in DMHA referrals during the first quarter of calendar years 2020 and 2021, with declining patterns in the periods between those quarters [9]. During this 2-year study period, there were variations in patient, clinical, and encounter type characteristics observed depending on whether the DMHA referrals included mindfulness and meditation alone, cognitive behavioral therapy alone, or a combination of both, and these results have previously been reported in the literature [9].

### Objectives

Optimal use of DMHAs requires both clinicians’ willingness to prescribe to patients and patients’ willingness to adopt and engage in their use. With limited information available about factors that influence the adoption and use of DMHAs in routine clinical practice, we aimed to explore patient and HCP perspectives that may affect referral, adoption, and use of DMHAs for improving emotional well-being and addressing subclinical, mild to moderate mental health conditions in real-world settings.

## Methods

### Design

A pilot study using cross-sectional surveys spanning a 4-month period (March 2022 to June 2022) was conducted to gather patient and HCP perceptions about their recent past end user experience with DMHAs. The overarching approach for this study was guided, in part, by diffusion of innovation theory [10], where adoption of a new device, practice, or idea is dependent on three factors: (1) perception of the innovation, (2) characteristics of those who may adopt the change, and (3) contextual factors.

Where applicable, the elements of cross-sectional survey design, institutional review board (IRB) considerations, survey development and pretesting, recruitment process and sample description, survey administration, response rates, and analysis as outlined in published guidance [11] were followed and are described herein. Both the patient and HCP survey instruments (Multimedia Appendices 1 and 2) are provided for the reader to assess content, length, and structure. Because patients were explicitly recruited, completed a consent form, and were sent a participant-specific survey link, duplicate entries were avoided.

### Ethical Considerations

The study was approved by KPMAS IRB (1761101). Survey responses were not linked to the electronic health record. A US $25 electronic gift card for patient participants and a US $50 electronic gift card for HCP participants were offered as an incentive if the surveys were completed. Participant-specific survey links were sent via email addresses but were stored independently from the final analytic survey dataset. Participant survey responses were not linked to gift card distribution. No personally identifiable information is included in the manuscript or Multimedia Appendices 1 and 2. All study data were stored on an internal, password-protected KPMAS server, which required dual authentication for access.

### Patient and HCP Samples

To identify an eligible pool of participants, KP HealthConnect electronic health records were used to identify adult patients (aged ≥18 y) who were referred to ≥1 DMHAs between April 2021 and December 2021 during a health care encounter. This time frame for evaluation was selected to not overlap with past internal evaluation efforts. All HCPs who had referred (ie, ordered) ≥1 DMHAs during a health care encounter between the implementation of the DMHA initiative (December 2019) and December 2021 comprised the eligible HCP pool of participants.

From the eligible participant pools, quota samples of 60 patients and 12 HCPs were set as a goal for this initial pilot work. For patients, a randomized block sampling design was used with repeated sampling blocks of 63 until the quota was fulfilled. The patient sample was stratified by each referral month (April 2021 through December 2021) to ensure representation across the entire observation period and include varying durations of potential use. The HCP sample was stratified to ensure representation from physicians and nonphysicians, and primary care, specialty, and behavioral health care HCPs in randomized blocks of 30 to fulfill the quota. Participant responses were deidentified and not linked to electronic health record data to encourage participation by ensuring confidentiality of responses. For both patient and HCP samples, random selection was used to optimize representation of the eligible participation pools.

### Measurements

As no standardized assessment existed due to the innovative nature of this work, clinician and patient surveys were developed de novo in accordance with accepted survey methods [12], while considering commonly accepted frameworks for technology evaluation (eg, American Psychiatric Association App Evaluation Model [13] and Technology Acceptance Model [14,15]) and other work related to the rating of mobile apps [16-18]. This was supplemented by internal evaluations from the national KP Project Chamai [8].

Once constructed, instruments were assessed for content validity by study investigators and modified via consensus discussions. Afterward, the surveys were programmed into REDCap (Research Electronic Data Capture; Vanderblit University) [19,20], which is a secure, Health Insurance Portability and Accountability Act (HIPAA)–compliant web-based application for administering electronic surveys. The instruments were iteratively refined vis-à-vis before testing within the research team and feedback from clinician colleagues. The final IRB-approved electronic versions of the instruments were distributed via email to the target patient and HCP recruitment pools derived from KP HealthConnect via participant-specific survey links.

The patient survey instrument is provided as Multimedia Appendix 1. Items were organized into the following three domains: (1) DMHA experience (ie, recall of DMHA referral from HCP, types of DMHAs used, extent of use, and reasons for initiating or continuing DMHA use; reasons for not starting or discontinuing DMHA use; and importance of DMHA attributes with respect to engagement, functionality, design, and information categories); (2) current emotional and mental health and wellness (ie, Patient Health Question 4 [PHQ-4] [21] and Subjective Happiness Scale [22]); and (3) respondent demographic information, including age, gender, education level, race or ethnicity, marital status, income sufficiency, English as a first language, general health status, health literacy [23], and confidence using a smartphone to manage emotional health and wellness.

The HCP survey instrument is provided as Multimedia Appendix 2. The questions were organized into the following three domains: (1) DMHA referrals (ie, types of DMHAs, how many DMHAs referred at 1 encounter, advantages and disadvantages of referring multiple DMHAs, and reasons for DMHA referral); (2) importance of DMHA attributes with respect to engagement, functionality, design, and information categories; and (3) respondent demographic information. Additional open-ended questions were used to allow HCPs to describe facilitators and barriers to DMHA referral and use as well as a typical patient referral to enhance discussion of the findings. Because the DMHA initiative had been implemented for >1 year at the time of survey, a description of the “typical” patient referral was requested rather than also requesting descriptions of the worst- and best-case scenarios. This approach also helped to minimize respondent burden.

For both survey instruments, questions were presented in a consistent order using a mix of yes or no, multiple selection, rank ordering, and Likert-type scale response sets. In contrast with the patient survey, open-ended questions were also used to gather a more detailed qualitative description of the HCP experience with DMHA referrals.

### Procedures and Survey Implementation

Patients selected from the eligible participant pool were initially contacted via a personal telephone call and, if interested, were sent a subsequent email invitation to participate in this closed survey. The email invitation included a participant-specific link to the electronic consent form and electronic survey distributed through REDCap [19,20]. The consent form was presented in a question and answer format that included the following: study title; contact information of the principal investigator and research associate; brief study overview; why the study was being conducted; what will happen when participating; how long participation will take; potential risks, discomforts, and benefits; incentives for participation; confidentiality and safeguarding of information; reasons for withdrawal by the investigator; rights of voluntary participation; contact information for questions; and a signature block acknowledging understanding and willingness to participate. Research staff attempted to contact eligible patients by phone on a maximum of 3 occasions, with each call attempt at least 3 days apart. If a patient was not reached, a voice message was left when possible. Internal administrative policy mandates that HCPs be initially contacted by email, rather than by telephone, to invite them to participate in the electronic survey distributed via REDCap [19,20]. The survey included an initial triage question to avoid surveying patients or HCPs who did not recall receiving or providing a DMHA referral. Participants had the opportunity to review and edit their responses before submitting the survey.

For each survey invitation sent, up to 3 weekly email reminders to complete the survey were sent to interested participants, as needed. Survey links were disabled 4 weeks after the initial email was sent if they were not completed. Participants had up to 7 days to complete the survey after initially opening the link before it was disabled.

### Analysis

Data were extracted from REDCap [19,20], organized and cleaned, and reported using descriptive statistics (ie, means, measures of variability, and frequencies and proportions depending on the level of measurement) to profile the patient and HCP survey responses. Rather than requiring fully completed surveys, analyses were conducted by question unless all items were necessary to calculate a specific scale result. Internal consistencies for the PHQ-4 [21] and Subjective Happiness Scale [22] were calculated to establish measurement reliability for these scales. A pairwise correlation between the PHQ-4 [21] and Subjective Happiness Scale [22] was calculated to further establish response consistency and reliability. Because each of the 4 DMHA attribute domains had a different number of attributes to rank (ie, engagement, 6/6, 100%; functionality, 5/5, 100%; design, 4/4, 100%; and information domains, 5/5, 100%), the rank-order scores within each domain were standardized to reflect a scale of 100 to simplify interpretation. To do this, individual rank-order scores for each attribute were first coded so that higher values reflected higher perceived importance. The recoded importance scores were then multiplied by (100/number of attributes in the domain) to create a scale where lower values reflected less importance and higher values reflected more importance with a ceiling value of 100. Analyses were performed using Stata (version 16.1; StataCorp LLC).

## Results

### Survey Response Rates: Patient Survey

There were 9525 patients referred to at least 1 DMHA between April 2021 and December 2021. Of those 9525 patients, 378 (3.97%) were randomly sampled from 6 blocks of 63 patients. A total of 119 patients agreed to participate and were sent a survey link. Of those 119 patients, 62 (52.1%) clicked on the emailed survey link and 58 (48.7%) completed the survey between March 2022 and June 2022, resulting in an effective response rate of 48.7% (58/119). Importantly, 24% (14/58) of the patients did not recall or report being referred to a DMHA and were removed from the analyses because the survey questions were intended to reflect user experience, resulting in a usable response rate of 37% (44/119).

### Survey Response Rates: HCP Survey

There were 410 HCPs who had referred at least 1 DMHA during a health care encounter between the implementation of the DMHA initiative (December 2019) and December 2021. Of the 410 HCPs, 60 (14.6%) from 2 blocks of 30 HCPs per block were invited to complete the survey. A total of 12 HCPs completed the survey between March 2022 and May 2022, fulfilling the quota sample with an effective response rate of 20%.

### Patient and HCP Characteristics

A total of 44 patient respondents completed the survey and reported having a DMHA referred to them by their HCP (Table 1). Of those 44 patient respondents, 32 (73%) were female participants, 32 (73%) had at least a 4-year college degree, 18 (41%) were Black or African American, and 17 (39%) were White. Mean age of the patient respondents was 42 (SD 14.08) years (29/44, 66%). Moreover, 27% (12/44) of the patient respondents screened positive for anxiety symptoms, and 23% (10/44) of the patient respondents screened positive for depression symptoms using items from the PHQ-4 [21]. Collectively, one-fourth (11/44, 25%) of the patient respondents reported a moderate to severe (≥6) PHQ-4 [21] overall score. The mean and median Subjective Happiness Scores [22] were 4.73 (SD 1.22) and 4.75 (IQR 3.75-5.75), respectively, and within the average range of 4.5 to 5.5 of other sample norms. Subjective Happiness Scores [22] were inversely correlated with PHQ-4 scores [21] (*r*=–0.44; *P*=.003; 43/44, 98%), indicating reliable survey completion.

**Table 1:** Patient demographic characteristics (N=44).

Characteristics		Values
**Age (y)**		
	Mean (SD; range)	42.21 (14.08; 21-77)
	n (%)	29 (66)
**Gender, n (%)**		
	Female	32 (73)
	Male	11 (25)
	Other	1 (2)
**Education level, n (%)**		
	High school graduate or General Educational Development test	1 (2)
	Some college or 2-year degree	11 (25)
	4-year college degree	10 (23)
	More than 4-year college degree	22 (50)
**Race or ethnicity, n (%)**		
	Asian	2 (5)
	Black or African American	18 (41)
	Hispanic or Latino	2 (5)
	Native Hawaiian or other Pacific Islander	1 (2)
	White	17 (39)
	>1 race or Hispanic ethnicity	3 (7)
	Prefer not to say	1 (2)
**Marital status, n (%)**		
	Divorced or separated	8 (18)
	Married	18 (41)
	Single, never married	17 (39)
	Widowed	1 (2)
**Self-reported general health**		
	Poor, n (%)	—^a^
	Fair, n (%)	13 (30)
	Good, n (%)	13 (30)
	Very good, n (%)	14 (32)
	Excellent, n (%)	4 (9)
	Mean (SD)	3.20 (0.98)
**English as a first language, n** **(%)**		
	Yes	38 (86)
	No	6 (14)
**Income sufficient to meet basic needs, n** **(%)**		
	Yes	42 (95)
	No	2 (5)
**Confidence completing medical forms by themself, n** **(%)**		
	Extremely	37 (84)
	Quite a bit	5 (11)
	Somewhat	2 (5)
**Need help from someone to read hospital materials, n** **(%)**		
	None of the time	39 (89)
	A little of the time	3 (7)
	Some of the time	2 (5)
**Problems learning about medical condition because of difficulty understanding written information, n** **(%)**		
	None of the time	36 (82)
	A little of the time	7 (16)
	Some of the time	1 (2)
**Confidence using smartphone to manage mental health and wellness, n** **(%)**		
	Extremely	29 (66)
	Quite a bit	11 (25)
	Somewhat	2 (5)
	A little bit	1 (2)
	Not at all	1 (2)
**Global self-rating of overall mental or emotional health**		
	Poor, n (%)	1 (2)
	Fair, n (%)	19 (43)
	Good, n (%)	12 (27)
	Very good, n (%)	7 (16)
	Excellent, n (%)	4 (9)
	Missing, n (%)	1 (2)
	Mean (SD); n (%)	2.86 (1.04); 43 (98)
**PHQ-4** ^b^ **anxiety and depression score, n** **(%)**		
	Normal (0-2)	21 (48)
	Mild (3-5)	11 (25)
	Moderate (6-8)	7 (16)
	Severe (9-12)	4 (9)
	Missing	1 (2)
**Anxiety (first 2 questions of PHQ-4), n** **(%)**		
	Yes (≥3)	12 (27)
	No (<3)	31 (70)
	Missing	1 (2)
**Depression (last 2 questions of PHQ-4), n** **(%)**		
	Yes (≥3)	10 (23)
	No (<3)	33 (75)
	Missing	1 (2)
**Assumes personal responsibility for their own mental and emotional health and well-being, n** **(%)**		
	Neither agree nor disagree	1 (2)
	Agree	10 (23)
	Agree strongly	31 (70)
	Missing	2 (5)
**Subjective Happiness Score**		
	Mean (SD)	4.73 (1.22)
	Median (IQR)	4.75 (3.75-5.75)
	Values, n (%)	43 (98)
	Scale Cronbach α	0.79

^a^No observations reported.

^b^PHQ-4: Patient Health Question 4.

A total of 12 HCPs completed the survey (Table 2). Mean age of the HCP respondents was 46 (SD 7.75) years (11/12, 92%), and 92% (11/12) of the HCPs were female. The mean number of years since completing training was 14.3 (SD 9.94) years (10/12, 83%). Of the 12 HCP respondents, 7 (58%) were physicians, with the remainder (n=5, 42%) either a licensed psychologist or clinical social worker. All HCP respondents personally tried at least 1 mindfulness or meditation DMHA, but only 42% (5/12) of the respondents personally tried at least 1 cognitive behavioral therapy DMHA.

**Table 2:** Health care professional (HCP) demographic characteristics (n=12).

Characteristic			Values
**Age (y)**			
	Mean (SD; range)	46 (7.75; 34-63)	
	n (%)	11 (92)	
**Gender, n (%)**			
	Female	11 (92)	
	Male	1 (8)	
**Highest level of training, n (%)**			
	Licensed clinical social worker	4 (33)	
	Licensed psychologist	1 (8)	
	Physician	7 (58)	
**Primary area of practice, n (**%)			
	Behavioral health	6 (50)	
	Primary care	6 (50)	
**Age range of patients primarily served (y), n (%)**			
	<19	4 (33)	
	19 to <45	8 (67)	
	45 to <65	11 (92)	
	>65	7 (58)	
**Digital mental health apps tried by HCP, n (%)**			
	Mindfulness and meditation apps	12 (100)	
	Cognitive behavioral therapy apps	5 (42)	
**Years since completing clinical training**			
	Mean (SD	14.3 (9.94; 4-33)	
	n (%)	10 (83)	

### DMHA Use and Referral

Approximately 73% (32/44) of the patient respondents recalled being referred to only 1 DMHA. Furthermore, 91% (40/44) of the patient respondents reported referral to 1 of the 3 mindfulness and meditation DMHAs more commonly, with only 25% (11/44) of the respondents recalling referral to 1 of the 3 cognitive behavioral DMHAs (result not reported in Table 3). Approximately 89% (39/44) of the patient respondents reported ever using at least 1 DMHA, and 61% (27/44) of the respondents reported DMHA use for ≤6 months after referral at the time of the survey. Of the 16 (36%) patient respondents who reported current use (ie, within the past 30 days), 75% (12/16) used the DMHA once daily to once weekly (result not reported in Table 3). Overall, 82% (36/44) of the patient respondents reported that DMHAs were at least somewhat helpful for improving mental and emotional health. Most common self-reported reasons for being referred to the DMHA included anxiety or panic control (25/44, 57%), mindfulness or meditation (15/44, 34%), sleep improvement (15/44, 34%), mood management (14/44, 32%), coping with specific issues such as grief and trauma (14/44, 32%), and stress reduction (13/44, 30%).

**Table 3:** Patient-reported digital mental health application (DMHA) referral and use characteristics (N=44).

Characteristic		Respondents, n (%)
**If referred by a health care professional, number of different DMHAs suggested**		
	1	32 (73)
	2	8 (18)
	3	4 (9)
**DMHA ever (ie, current or previous) use**		
	Yes	39 (89)
	No	5 (11)
**Duration of use for at least 1 DMHA since referral** **(months)**		
	<3	17 (39)
	4-6	10 (23)
	7-9	4 (9)
	10-12	1 (2)
	>12	7 (16)
	Missing	5 (11)
**Reasons for referral**		
	Anxiety or panic control	25 (57)
	Sleep improvement	15 (34)
	Mindfulness and meditation	15 (34)
	Coping with a specific issue (eg, grief, life changes, trauma, and relationships)	14 (32)
	Mood management (eg, depression and happiness)	14 (32)
	Stress reduction	13 (30)
	Personal well-being	7 (16)
	Balancing intense emotions (eg, anger)	6 (14)
	Chronic pain management	2 (5)
	Building resilience	1 (2)
	Parenting and caregiving	1 (2)
	Substance abuse (eg, nicotine, drug, or alcohol recovery)	1 (2)
**Helpfulness of the DMHAs for improving mental and emotional health and well-being**		
	Not very helpful	4 (9)
	Somewhat helpful	17 (39)
	Very helpful	14 (32)
	Extremely helpful	5 (11)
	Do not know	1 (2)
	Missing	3 (7)

Approximately half (5/12, 42%) of the HCP respondents ordered >1 DMHA at an encounter, with mindfulness and meditation DMHAs being more commonly referred (12/12, 100%) than cognitive behavioral therapy–based DMHAs (9/12, 75%; Table 4). Open-ended text responses from HCPs revealed that patient choice, variety, and increased options were advantages to referring >1 DMHA at a time. However, HCPs recognized that this strategy may provide too much information and may be overwhelming for the patient when >1 DMHA is referred at an encounter. Most common reasons for DMHA referral included anxiety and panic control (8/12, 67%), mood management (7/12, 58%), mindfulness and meditation (6/12, 50%), stress reduction (4/12, 33%), sleep improvement (3/12, 25%), and balancing intense emotions (3/12, 25%).

**Table 4:** Health care professional (HCP)–reported digital mental health application (DMHA) referral or ordering characteristics (n=12).

Characteristic			Respondents, n (%)
Ever ordered a DMHA			12 (100)
**DMHAs ordered by a HCP**			
	Mindfulness and meditation apps	12 (100)	
	Cognitive behavioral therapy apps	9 (75)	
**Number of DMHAs typically ordered at 1 encounter**			
	1	7 (58)	
	2	5 (42)	
**Advantages of ordering >1 DMHA at an encounter (n=5)**			
	Having the app options for which works best for individual patient struggles	1 (8)	
	At least they will download and use 1 of them	1 (8)	
	Patient can choose the features from each app that are most helpful	1 (8)	
	More options	1 (8)	
	Greater variety	1 (8)	
**Disadvantages of ordering >1 DMHA at an encounter (n=5)**			
	Can create confusion	1 (8)	
	May be too much information for the patient at 1 time	1 (8)	
	Some patients may feel overwhelmed if they get >1 suggestion	1 (8)	
	More work for the member to figure out >1 app	1 (8)	
	Confusion as to which one to choose	1 (8)	
**Conditions most commonly associated with DMHA referral**			
	Anxiety or panic control	8 (67)	
	Mood management (eg, depression and happiness)	7 (58)	
	Mindfulness and meditation	6 (50)	
	Stress reduction	4 (33)	
	Sleep improvement	3 (25)	
	Balancing intense emotions (eg, anger)	3 (25)	
	Chronic pain management	1 (8)	
	Coping with a specific issue (eg, grief, life changes, trauma, and relationships)	1 (8)	
	Substance use (eg, nicotine, drug, or alcohol recovery)	1 (8)	

### Perceived Importance of DMHA Attributes

Within each DMHA attribute domain, the most important DMHA attributes that patient respondents reported (Table 5) included being fun and interesting to use (Importance Score [IS]=68.9), having personalized settings (IS=68.3), and content that appeals to personal preferences (IS=68.3) for the engagement domain; ease in learning how to use (IS=84.4) and clear menu labels, icons, and instructions (IS=69.4) for the functionality domain; visual appeal (IS=75.0) and narrator voice (IS=66.0) for the design domain; and having well-written, goal- and topic-relevant content (IS=80.0) and credible and legitimate sources (IS=67.9) for the information domain.

**Table 5:** Patient- and health care professional (HCP)–reported importance of digital mental health app features and characteristics.

Feature and characteristic		Patient Importance Score	HCP Importance Score
**Engagement^a^, n (%)**		30 (100)	9 (100)
	Fun, entertaining, or interesting to use, mean (SD)	68.9 (26.5)	61.1 (22.1)
	Settings can be personalized (eg, reminders, notifications, sound, content, challenges and goal setting, and sharing options), mean (SD)	68.3 (29.5)	75.9 (22.2)
	Content (eg, visuals, language, and design) appeals to my personal preferences, mean (SD)	68.3 (22.9)	75.9 (30.2)
	Allows user input, provides feedback, and contains prompts, mean (SD)	55.0 (25.2)	51.9 (29.4)
	Allows you or user to be contacted by a mental health HCP if needed, mean (SD)	44.4 (27.5)	38.9 (23.6)
	Provides availability of a coach, mean (SD)	45.0 (28.8)	46.3 (28.6)
**Functionality^a^, n (%)**		36 (100)	11 (100)
	Functions fast, mean (SD)	67.8 (23.6)	49.1 (27.4)
	Easy to learn how to use, mean (SD)	84.4 (22.5)	94.6 (9.3)
	Clear menu labels, icons, and instructions, mean (SD)	69.4 (17.6)	70.9 (16.4)
	Taps, swipes, pinches, and scrolls (movement) that make sense, mean (SD)	50.0 (19.4)	41.8 (20.9)
	Available in multiple languages, mean (SD)	28.3 (21.6)	43.6 (25.0)
**Design^a^, n (%)**		39 (100)	10 (100)
	Arrangement and size of buttons, icons, menus, and content on the screen, mean (SD)	49.4 (27.2)	82.5 (20.6)
	Quality and resolution of the app graphics used for buttons, icons, menus, and content, mean (SD)	59.6 (22.7)	60.0 (26.9)
	Visually appealing, mean (SD)	75.0 (22.2)	40.0 (21.1)
	Narrator voice (eg, gender, tone, and accent), mean (SD)	66.0 (33.2)	67.5 (29.0)
**Information^a^, n (%)**		38 (100)	12 (100)
	Content is well written and relevant to the stated goals or topics, mean (SD)	80.0 (22.8)	81.7 (24.8)
	Quantity of the information is comprehensive but concise, mean (SD)	56.3 (20.7)	65.0 (21.1)
	Duration of the app sessions, mean (SD)	52.1 (27.7)	48.3 (30.1)
	Visual information (eg, charts, graphs, images, and videos) used to explain concepts is clear and logical, mean (SD)	43.7 (23.2)	51.7 (26.2)
	Information within comes from a legitimate or credible source, mean (SD)	67.9 (32.1)	78.3 (28.9)

^a^Rank order responses for items within each domain (ie, engagement, functionality, design, and information). Responses were coded and rescaled to 100, where lower values indicate less importance and higher values indicate more importance.

HCP respondents reported some similar but also some different DMHA features and ranking variability within each domain as being the most important (Table 5). They included having personalized settings (IS=75.9) and content that appeals to personal preferences (IS=75.9) for the engagement domain; ease of learning how to use (IS=94.6) and clear menu labels, icons, and instructions (IS=70.9) for the functionality domain; arrangement and size of buttons, icons, menus, and content on the screen (IS=82.5) and narrator voice (IS=67.5) for the design domain; and having well-written, goal- and topic-relevant content (IS=81.7) and credible and legitimate sources (IS=78.3) for the information domain. In open-ended text responses, HCPs also suggested other important features that included the ability to journal or take notes; having a daily mood tracker; substance use– or chemical dependency–specific application; topical content that can be upgraded; user ability to download information or audio books; and having information that is relevant and specific, rather than vague, general recommendations.

### HCP-Reported Barriers to DMHA Use

From open-ended responses, the most commonly reported barriers observed by HCPs included difficulty downloading and registering the DMHA; lack of patient motivation, self-direction, and consistent use; time constraints for both patient and HCPs; and follow-up about DMHA use and effectiveness.

### Typical Patients for Whom DMHAs Are Referred

Using open-ended text responses, HCPs described “typical patients” commonly referred to DMHAs based on perceived need, technical capability, and common medical conditions. As summarized, perceived need factors included (1) healthy adults who are not yet ready for psychiatry or psychotherapy but would benefit from self-awareness and coaching on their own time; (2) patients looking for ways to understand their own reactions better or wanting to gain insight into their own thought processes as a means for improving anxiety, stress, or depression; and (3) patients needing help with learning cognitive behavioral therapy skills and grounding exercises to help regulate emotion. Perceived technical capability included (1) patients who are smartphone or technology savvy, who already incorporate technology and apps into their lifestyle (eg, Fitbit and music apps), and who use them as part of their entertainment; (2) adolescents and younger patients who are more likely to use DMHAs; and (3) patients who have time to actually use the DMHA and are open to try other resources. In their own words, the top 10 most common medical conditions for which HCPs referred DMHAs included the following: (1) stress, anxiety (mild to severe), and generalized anxiety disorder; (2) depression and major depressive disorder; (3) emotional dysregulation; (4) distorted and negative thinking; (5) posttraumatic stress disorder; (6) sleep disturbance and insomnia; (7) multiple somatic symptoms; (8) traumas from the past; (9) unhappiness; and (10) chronic pain.

## Discussion

### Overview

This study describes concurrent patient and HCP perspectives within an integrated health system that may affect referral, adoption, and use of DMHAs for improving emotional well-being and mental health conditions in the same practice environment. Importantly, the results reinforce the necessity to identify and tailor individual patient needs and preferences with the most appropriate DMHA to ensure successful engagement and sustainable use. These themes are supported by prior research [24] that identified 16 constructs that may influence user engagement with DMHAs; these constructs are grouped by user (ie, demographics, personal traits, mental health status, beliefs, mental health technology experience, and integration into life), program (ie, type of content, perceived fit, perceived usefulness, level of guidance, social connectedness, and impact of intervention), and technology environment (ie, technology-related factors, privacy and confidentiality, social influence, and implementation) [24]. Many of these constructs manifest themselves in this research and are highlighted in the subsequent sections.

### Facilitating Patient Uptake and Sustained Use

Both low and sustained engagement with DMHAs have been recognized as a barrier to successful use [24,25]. In this study, approximately three-fourths (44/58, 76%) of the patients recalled or reported being referred to at least 1 DMHA, suggesting that approximately one-fourth (14/58, 24%) of the patients referred to DMHAs did not engage with them despite documented referral. Although a majority of patients (39/44, 89%) who recalled being referred to a DMHA reported at least some use, only a minority (12/44, 27%) reported >6 months of continued use. It is likely that the time between referral and the survey may have reduced the reported duration of use, making this a conservative estimate, and despite this limitation, the levels of engagement, ever use, and sustained use appear to be higher than those reported in previous research [26,27]. Although only 36% (16/44) of the patient respondents reported current DMHA use, 75% (12/16) of those who did also reported once daily to once weekly use. This finding reinforces the notion that while DMHAs are not a viable or ideal therapeutic solution for everyone, they may have an important role in a select group of patients who routinely use them. Moreover, the ideal or preferred duration of use will likely depend on the condition for which the DMHA is being used. Given the wide array of conditions for DMHA use reported in this pilot research, it is not possible to determine optimal duration for each, and this remains an important area for future research.

Collectively, these findings suggest that initial engagement and sustained use of DMHAs was modest even when an organization encourages and supports their use. If patients are to realize the full benefit of DMHA use, health system interventions that promote and increase identification of patients most likely to engage and sustain DMHA use should be a focus of future efforts. Importantly, 82% (36/44) of the patient respondents reported some degree of helpfulness from the DMHA and >90% (41/44) of the patient respondents agreed or strongly agreed with assuming personal responsibility for their own mental and emotional health and well-being. This aligns with prior research suggesting that DMHAs could be empowering and supportive of self-management [28].

It is recognized that guidance and training facilitate DMHA engagement [24]. On the basis of their own experience, HCP respondents offered practical advice to facilitate successful initiation and sustainable use of DMHAs. It is critical that HCPs invest time, not only at initiation of referral but also during ongoing care, to raise awareness and reinforce why and how DMHA use is an important component of their treatment plan and to encourage engagement. Simply referring a patient to a DMHA with limited explanation is likely insufficient.

It has been previously reported that HCPs who use DMHAs themselves are more likely to use them in practice [29]. In this study, all HCP respondents (12/12, 100%) reported use of a mindfulness and meditation DMHA, but less than half (5/12, 42%) reported use of a cognitive behavioral therapy DMHA. To facilitate use, HCPs recommended using the DMHAs themselves; describing their own experience with the DMHAs; and providing a subsequent show-and-tell with the patient, if comfortable. Assisting the patient with downloading the DMHAs, walking them through how to use it, demonstrating the different features and functionality, and showing them how to find clinically relevant content was also helpful. Collectively, these actions may overcome technology literacy barriers. While these best practices are ideal, time, resource, and reimbursement limitations may foil their implementation. HCPs may become fatigued if they have to perform this for all their patients, which could lead them to stop integrating DMHAs into care if the process is too burdensome. Therefore, alternative solutions and resources to facilitate implementation must be created for sustainability purposes.

Assessing patient interest and highlighting an aspect of the DMHA that aligns with the patient’s area of interest, describing the purpose and value of the DMHA, and ensuring the patient understands that it is an adjunct to in-person therapy and an opportunity to reinforce skills learned in their in-person session may also be beneficial. To support goal setting, HCPs suggested integrating the DMHA into the treatment plan by explaining rationale (eg, mindfulness to decrease anxiety) and indicating that it will be discussed at the next session. Finally, HCP respondents recommended in-person practice using the DMHA and setting reminders for the patient to use the DMHA between sessions, while reassuring ease of use and reinforcing that it does not need to be used daily to further facilitate engagement.

### Aligning Patient Preferences and Needs

While patients and HCPs often prioritize the importance of many key DMHA attributes similarly, it is important for HCPs to recognize that a “one-size-fits-all” approach is not ideal [24]. HCPs must identify unique patient preferences rather than using preferences based on their own user experience. In the engagement domain, patients placed highest importance on DMHAs that are fun or interesting to use, whereas HCPs placed less importance on this attribute. Consistent with past reports [24], HCPs placed highest importance on personalized settings and content in the engagement domain, with patients reporting these attributes with second highest importance. With respect to the design domain, patients placed highest importance on visual appeal, while HCPs reported the arrangement and size of buttons, icons, and menus to be most important. The fact that both patients and HCPs in this study reported past or current experience with DMHA use underscores the credibility of these findings related to preferences.

Importantly, previous research recognized that engagement was facilitated with an “appropriate” amount of credible content [24]. In this study, both patients and HCPs reinforced the importance of this attribute by placing it as the highest priority in the information domain. Both patients and HCPs assigned the highest importance to the ease-of-use attribute in the functionality domain. Interestingly, both patients and HCPs assigned the least importance to the DMHA attribute that allows them to be contacted by a mental health HCP, if needed, suggesting a desire for privacy. This observation is supported by the literature that DMHA engagement was improved when there was reassurance that the DMHA provided privacy and anonymity [24].

Finally, patients and HCPs expressed a wide array of patient needs for which the DMHA was referred. Given that it is uncommon for a DMHA to excel in every clinical domain, 42% (5/12) of the HCPs reported that they may order >1 DMHA at an encounter, and 27% (12/44) of the patients indicated that >1 DMHA was recommended by a HCP. These findings underscore the importance of understanding patient needs and preferences and then tailoring the referral of a DMHA to avoid overwhelming the patient and creating potential confusion.

Considering the varying preferences, HCPs must identify the attributes and content that are important and necessary to align the DMHA with each patient’s needs. With a wide array of DMHAs available, HCPs and health systems must develop a decision support system that facilitates alignment of patient clinical needs and their preferred attributes with the best-suited DMHA.

### Limitations

While this research is unique in incorporating both patient and HCP perspectives of active DMHA users in a real-world setting, it does have limitations. The small sample, low response rate, and focus within an integrated health system limit generalizability to other practice settings. This research was designed to minimize respondent identification and, therefore, relied on participant self-reported responses, with no linkage to medical records. As such, some variables incurred a number of missing observations (eg, age), but for the most part, missingness was limited. Importantly, the patient sample reflected a slightly older age (42 vs 39 y), similar distributions of White and Black patients (17/44, 39% and 18/44, 41% vs 3473/9525, 36.5% and 3702/9525, 38.9%, respectively), fewer Hispanic patients (2/44, 5% vs 1028/9525, 10.8%), and similar distribution of female patients (32/44, 73% vs 6754/9525, 70.9%) compared to the overall referral pool of 9525 eligible patients during the observation period between April 2021 and December 2021. The HCP sample included a smaller percentage of physicians (7/12, 58% vs 279/410, 68%) and increased percentage of behavioral health care HCPs (6/12, 50% vs 168/410, 41%) than the total pool of 410 eligible HCPs who had referred at least 1 DMHA during a health care encounter between the implementation of the DMHA initiative (December 2019) and December 2021. Despite recruitment, there were no specialty physicians included in the HCP sample. Moreover, the samples were limited to respondents with English language proficiency. In the absence of an ideal survey instrument for purposes of this research, many items were developed de novo but were constructed using acceptable assessment frameworks, evaluated for content validity, and piloted within the study team before use. Given the voluntary nature of this research, a completeness check was not required. However, response consistency was assessed and confirmed by comparing PHQ-4 [21] and Subjective Happiness Scores [22]. Future work should include both HCP and patient end user feedback in instrument design and data collection. Finally, this research mainly focuses on implementation and does not evaluate clinical impact that should be incorporated and studied in future work. Importantly, this research provides a start to a more comprehensive effort to inform clinical decision support and workflow design initiatives that hope to optimize DMHA referral, engagement, sustained use, and clinical effectiveness.

### Conclusions

To effectively implement DMHA engagement and sustained use in real-world practice, health systems must develop support systems to identify, align, and tailor individual patient needs and preferences with the most appropriate DMHA. Future research should focus on design of a HCP decision guide that addresses clinician and patient awareness, knowledge, and common misperceptions regarding DMHAs; optimizes alignment of patient need, preferences, and DMHA attributes; encourages engagement and fidelity to DMHA use for widespread adoption across health systems; and subsequently evaluates the impact on mental health outcomes.

## Appendix

Multimedia Appendix 1 Patient survey.

Multimedia Appendix 2 Health care professional survey.

## Abbreviations

DMHA: digital mental health application
HCP: health care professional
HIPAA: Health Insurance Portability and Accountability Act
IRB: institutional review board
IS: Importance Score
KP: Kaiser Permanente
KPMAS: Kaiser Permanente Mid-Atlantic States
PHQ-4: Patient Health Question 4
REDCap: Research Electronic Data Capture

## Data Availability

The datasets generated and analyzed during this study are not publicly available due to their proprietary nature. Moreover, the informed consent documents explicitly stated that no individual participant-level data would be disclosed and that only summary-level results would be reported in scholarly works.
