# Supplementary material for: Patient and Health Care Professional Perspectives About Referral, Self-Reported Use, and Perceived Importance of Digital Mental Health App Attributes in a Diverse Integrated Health System: Cross-Sectional Survey Study
Source: JMIR Form Res. 2024 Nov 15;8:e59831. doi: 10.2196/59831 (PMC11607546; doi:10.2196/59831)
Supplement: Multimedia Appendix 1 [file formative_v8i1e59831_app1.pdf]

Digital Mental Health and Wellness Apps

WELCOME TO THE SURVEY ON KAISER PERMANENTE’S DIGITAL MENTAL AND EMOTIONAL HEALTH AND WELLNESS APPS!

This survey will display best on a computer. If you are having trouble completing the survey on a mobile device, you may consider completing the survey on a computer. If you choose to take the survey on a mobile device, turning your phone horizontally may help you to view the questions better.

You may move forward and backward through this survey by clicking the "Next Page" or "Previous Page" buttons. You may return to any page to review and/or change your answers before submitting your survey. Your answers cannot be changed once you submit the survey.

If you cannot complete the survey at one time, you can select "Save & Return Later" at the bottom on the screen. A code will be provided and remain active so you can return to complete your survey.

Thank you for your participation!

YOUR EXPERIENCE WITH KAISER PERMANENTE’S DIGITAL MENTAL AND EMOTIONAL HEALTH AND WELLNESS APPS

Kaiser Permanente recently began offering some members apps (on smartphone, tablet, and computer) to help with mental and emotional health and wellness. The questions in this section are about your experience with the referral process and use of the app(s).

Please note:

For questions in this survey, Mental and Emotional Health and Wellness apps will be referred to as mental health apps. All survey questions are optional. You are not required to complete questions that you do not want to answer.

SECTION 1: Experience with apps for Mental and Emotional Health and Wellness

Did your Kaiser Permanente provider suggest that you try any of the following apps: Calm, Headspace, myStrength, Whil, SilverCloud, or Thrive?

◯ Yes
◯ No
◯ Don't know/Not sure

Which app(s) did your provider suggest? Check all that apply.

☐ Calm
☐ Headspace
☐ myStrength
☐ Whil
☐ SilverCloud
☐ Thrive
☐ Don't know/Not sure

Which of the following reasons best describes why your provider suggested you try [app_referred:checked]? (Check up to 3 reasons.)

☐ Anxiety or panic control
☐ Balancing intense emotions (e.g., anger, etc.)
☐ Building resilience
☐ Chronic pain management
☐ Coping with a specific issue (e.g., grief, life changes, trauma, relationships etc.)
☐ Mindfulness/meditation
☐ Mood management (e.g., depression, happiness, etc.)
☐ Parenting and caregiving
☐ Personal wellbeing
☐ Pregnancy or early parenting
☐ Sleep improvement
☐ Stress reduction
☐ Substance Use (e.g., Nicotine, drug, or alcohol recovery)
☐ Other (Specify): ______

Which of the following reasons best describes why your provider suggested you try one of the mental health app(s)? (Check up to 3 reasons.)

- ☐ Anxiety or panic control
- ☐ Balancing intense emotions (e.g., anger, etc.)
- ☐ Building resilience
- ☐ Chronic pain management
- ☐ Coping with a specific issue (e.g., grief, life changes, trauma, relationships etc.)
- ☐ Mindfulness/meditation
- ☐ Mood management (e.g., depression, happiness, etc.)
- ☐ Parenting and caregiving
- ☐ Personal wellbeing
- ☐ Pregnancy or early parenting
- ☐ Sleep improvement
- ☐ Stress reduction
- ☐ Substance Use (e.g., Nicotine, drug, or alcohol recovery)
- ☐ Other (Specify): ______

Please indicate your usage of each mental health app below:

	**Currently using (Within last 30 days)**	**Previously used (Not within last 30 days)**	**Did not use**
Calm	○	○	○
Headspace	○	○	○
myStrength	○	○	○
SilverCloud	○	○	○
Thrive	○	○	○
Whil	○	○	○

Which of the following reason(s) motivated you to start using one or more mental health app(s)? Check all that apply.

- ☐ My provider suggested that I try it.
- ☐ I heard about the app before and wanted to try it.
- ☐ I tried the app already and want to continue using it.
- ☐ I already practice mindfulness/meditation and wanted to try it with the app.
- ☐ I felt it would help me take better care of myself.
- ☐ I liked having it as an additional coping tool.
- ☐ It was free.
- ☐ It seemed easy to use.
- ☐ I was curious about the app.
- ☐ Not sure/no particular reason.
- ☐ Other. Please explain: ______

Which of the following reason(s) encourages you to continue using one or more mental health apps? Check all that apply.

- ☐ The app has been easy to use.
- ☐ The app has been helpful (e.g., better sleep, reduced stress, improved coping skills, etc.).
- ☐ The app provides useful information.
- ☐ The app provides notifications and reminders.
- ☐ The app is part of my self-care routine.
- ☐ The app is free.
- ☐ I like the look and feel of the app.
- ☐ I like what I'm learning from the app.
- ☐ Other friends/family/community/coworkers use the app.
- ☐ My provider encourages my use of the app.
- ☐ Not sure/no particular reason.
- ☐ Other. Please explain: ______

Which of the following reason(s) discouraged you from starting to use one or more mental health app(s)? Check all that apply.

☐ I wanted human interaction rather than an app.
☐ I am already in treatment for stress, depression, or anxiety and don't see the need for an app.
☐ I was concerned about the cost of the app.
☐ I was concerned about data security or privacy.
☐ I didn't want to spend too much time on my cell phone.
☐ I already practice mindfulness/meditation without the app.
☐ It would be embarrassing to have an app like this on my phone.
☐ Using the app seemed to go against my religion/beliefs.
☐ I had questions about the app but couldn't find someone to answer them.
☐ I don't feel comfortable with technology.
☐ I don't have reliable Wi-Fi/wireless connectivity.
☐ I don't have time to set up/learn how to use the app
☐ I was unable to download the app.
☐ I don't think I have a problem with stress, depression, or anxiety.
☐ Not sure/no particular reason.
☐ Other. Please explain: ______

Which of the following reason(s) caused you to stop using one or more mental health app(s) you were referred? Check all that apply.

☐ I was having technical difficultly using the app.
☐ I started feeling better after using the app and no longer needed it.
☐ I was spending too much time and effort using the app.
☐ I didn't find the content of the app interesting.
☐ I was not feeling any better when using the app.
☐ I ran out of storage on my phone.
☐ I was concerned about the cost of the app.
☐ I didn't like the physical features of the app(e.g. the way the app looked/sounded).
☐ The app was disruptive/annoying.
☐ No one followed up to check if the app was working for me.
☐ The app didn't accommodate for my disability (e.g., sight or hearing impairment).
☐ I didn't have the time to use the app.
☐ It felt like another responsibility.
☐ Not sure/no particular reason.
☐ Other. Please explain: ______

App Usage

Since you were referred to [app_referred], how long have you been using at least one mental health app?

- ◯ < 3 months
- ◯ 4-6 months
- ◯ 7-9 months
- ◯ 10-12 months
- ◯ More than 12 months

On average, how often have you used the following mental health app(s) in the past month?

	At least once a day	At least once a week	At least once in the past month	Did not use in past month	Don't Know
Calm	◯	◯	◯	◯	◯
Headspace	◯	◯	◯	◯	◯
myStrength	◯	◯	◯	◯	◯
Whil	◯	◯	◯	◯	◯
SilverCloud	◯	◯	◯	◯	◯
Thrive	◯	◯	◯	◯	◯

Of the following mental health app(s) that you either currently or previously used, how would you rate your overall experience with it?

	1-Poor	2	3	4	5-Excellent
Calm	◯	◯	◯	◯	◯
Headspace	◯	◯	◯	◯	◯
myStrength	◯	◯	◯	◯	◯
Whil	◯	◯	◯	◯	◯
SilverCloud	◯	◯	◯	◯	◯
Thrive	◯	◯	◯	◯	◯

For each group of features, rank the importance of each feature for you when you use a mental health app. You can only use each number once within a group of features. Therefore, no two features should have the same rank.

App Engagement (Please read all choices within the section before making your selections. Use each rating number only once.)

	1-Most Important	2	3	4	5	6-Least Important
Fun, entertaining or interesting to use	○	○	○	○	○	○
Settings can be personalized (e.g., reminders, notifications, sound, content, challenges and goal setting, sharing options, etc.)	○	○	○	○	○	○
Content (e.g., visuals, language, design) appeals to my personal preferences	○	○	○	○	○	○
Allows user input, provides feedback, and contains prompts	○	○	○	○	○	○
Allows you to be contacted by a mental health provider if needed	○	○	○	○	○	○
Provides availability of a coach	○	○	○	○	○	○

App Functionality (Please read all choices within the section before making your selections. Use each rating number only once.)

	1-Most Important	2	3	4	5-Least Important
Functions fast	○	○	○	○	○
Easy to learn how to use	○	○	○	○	○
Clear menu labels, icons, and instructions	○	○	○	○	○
Taps, swipes, pinches, scrolls (movement) that make sense	○	○	○	○	○
Available in multiple languages	○	○	○	○	○

App Design (Please read all choices within the section before making your selections. Use each rating number only once.)

	1-Most Important	2	3	4-Least Important
Arrangement and size of buttons, icons, menus and content on the screen	◯	◯	◯	◯
Quality/resolution of the app graphics used for buttons, icons, menus and content	◯	◯	◯	◯
Visually appealing	◯	◯	◯	◯
Narrator voice (e.g., gender, tone, accent, etc.)	◯	◯	◯	◯

App Information (Please read all choices within the section before making your selections. Use each rating number only once.)

	1-Most Important	2	3	4	5-Least Important
Content is well written, and relevant to the stated goals/topics	◯	◯	◯	◯	◯
Quantity of the information is comprehensive but concise	◯	◯	◯	◯	◯
Duration of the app sessions	◯	◯	◯	◯	◯
Visual information (e.g., charts, graphs, images, videos) used to explain concepts is clear and logical	◯	◯	◯	◯	◯
Information within comes from a legitimate or credible source	◯	◯	◯	◯	◯

Is there anything else you would like to share about how the app(s) was introduced and offered to you or your experience with using the app(s)?

How helpful are the app(s) for improving your mental and emotional health and well-being?

◯ Not at All Helpful
◯ Not Very Helpful
◯ Somewhat Helpful
◯ Very Helpful
◯ Extremely Helpful
◯ Don't know

SECTION 2: Current Mental Emotional Health and Wellness

Recognizing that your mental or emotional health can vary over time, in general, how would you rate your overall mental or emotional health?

◯ Poor
◯ Fair
◯ Good
◯ Very Good
◯ Excellent

Over the last 2 weeks, how often have you been bothered by the following problems?

	Not at All	Several Days	More Than Half the Days	Nearly Every Day
Feeling nervous, anxious or on edge	◯	◯	◯	◯
Not being able to stop or control worrying	◯	◯	◯	◯
Little interest or pleasure in doing things	◯	◯	◯	◯
Feeling down, depressed, or hopeless	◯	◯	◯	◯

Subjective Happiness Scale:

In general, I consider myself

Not a very happy person — A very happy person

1	2	3	4	5	6	7
◯	◯	◯	◯	◯	◯	◯

Compared with most of my peers, I consider myself

Less happy — More happy

1	2	3	4	5	6	7
◯	◯	◯	◯	◯	◯	◯

Some people are generally very happy. They enjoy life regardless of what is going on, getting the most out of everything. To what extent does this characterization describe you?

Not at all — A great deal

1	2	3	4	5	6	7
◯	◯	◯	◯	◯	◯	◯

Some people are generally not very happy. Although they are not depressed, they never seem as happy as they might be. To what extent does this characterization describe you?

Not at all — A great deal

1	2	3	4	5	6	7
◯	◯	◯	◯	◯	◯	◯

Please check your level of agreement with the following statement: When all is said and done, I am the person who is responsible for taking care of my mental and emotional health and well-being.

◯ Disagree Strongly
◯ Disagree
◯ Neither agree nor disagree
◯ Agree
◯ Agree Strongly

SECTION 3: Some Questions About You

Age in years (as of today)

__________________________ (years)

Gender

◯ Female
◯ Male
◯ Other
◯ Prefer not to say

What is the highest grade or level of school you have completed?

◯ 8th grade or less
◯ Some high school, but did not graduate
◯ High school graduate or GED
◯ Some college or 2-year degree
◯ 4-year college degree
◯ More than 4-year college degree
◯ Prefer not to say

How would you describe yourself? Check all that apply.

☐ White
☐ Native Hawaiian or Other Pacific Islander
☐ Black or African American
☐ Hispanic or Latino
☐ Native American (American Indian) or Alaska Native
☐ Asian
☐ Middle Eastern or North African
☐ Not listed, please specify: ______
☐ Prefer not to say

What is your marital status?

◯ Divorced or separated
◯ Married
◯ Single, never married
◯ Widowed
◯ Prefer not to say

Is English your first language?

◯ Yes
◯ No
◯ Prefer not to say

In general, would you say that your health is...

◯ Poor
◯ Fair
◯ Good
◯ Very Good
◯ Excellent

How confident are you filling out medical forms by yourself?

◯ Extremely
◯ Quite a bit
◯ Somewhat
◯ A little bit
◯ Not at all

How often do you have someone help you read hospital materials?

◯ All of the time
◯ Most of the time
◯ Some of the time
◯ A little of the time
◯ None of the time

How often do you have problems learning about your medical condition because of difficulty understanding written information?

◯ All of the time
◯ Most of the time
◯ Some of the time
◯ A little of the time
◯ None of the time

How confident are you in using your smartphone to manage your own mental and emotional health and wellness?

◯ Extremely
◯ Quite a bit
◯ Somewhat
◯ A little bit
◯ Not at all

Currently, is your income enough to meet your basic needs for food, housing, clothing, and medical care?

◯ Yes
◯ No

Please remember to review your responses and submit your survey!
