# Supplementary material for: Patient and Health Care Professional Perspectives About Referral, Self-Reported Use, and Perceived Importance of Digital Mental Health App Attributes in a Diverse Integrated Health System: Cross-Sectional Survey Study
Source: JMIR Form Res. 2024 Nov 15;8:e59831. doi: 10.2196/59831 (PMC11607546; doi:10.2196/59831)
Supplement: Multimedia Appendix 2 [file formative_v8i1e59831_app2.pdf]

YOUR EXPERIENCE WITH PRESCRIBING KAISER PERMANENTE'S DIGITAL OR ONLINE RESOURCES

Kaiser Permanente recently begun offering some members apps (on smartphone, tablet, and computer) to help with mental and emotional health and well-being. The questions in this section are about your experience when ordering or referring patients to the apps and your overall impressions of their use in the management of your patients' mental and emotional health and well-being.

Please note:
All survey questions are optional. You are not required to complete questions that you do not want to answer. For questions in this survey, Mental and Emotional Health and Wellness apps will be referred to as mental health apps.

Have you ever referred or ordered a mental health app (i.e., Calm, Headspace, myStrength, Whil, SilverCloud, or Thrive) for one of your patients?

- ○ Yes
- ○ No
- ○ Don't know/Not sure

Which of the following app(s) for mental and emotional health and wellness have you referred or ordered for your patients? (Check all that apply.)

- ❑ Calm
- ❑ Headspace
- ❑ myStrength
- ❑ Whil
- ❑ SilverCloud
- ❑ Thrive
- ❑ Don't know
- ❑ None of the above

When you decide to refer a patient to a mental health app, how many apps do you typically order at one encounter?

- ○ One
- ○ Two
- ○ Three or more

Please list the advantages and disadvantages of referring more than one mental health app at one time.

Advantages	Disadvantages

Of the following mental health apps, which have you most commonly referred or ordered? (Check up to two.)

- ❑ Calm
- ❑ Headspace
- ❑ myStrength
- ❑ While
- ❑ SilverCloud
- ❑ Thrive
- ❑ Don't know

For which of the following conditions do you most commonly refer mental health apps? (Check up to three.)

- ❑ Anxiety or panic control
- ❑ Balancing intense emotions (e.g., anger, etc.)
- ❑ Building resilience
- ❑ Chronic pain management
- ❑ Coping with a specific issue (e.g., grief, life changes, trauma, relationships etc.)
- ❑ Mindfulness/meditation
- ❑ Mood management (e.g., depression, happiness, etc.)
- ❑ Parenting and caregiving
- ❑ Personal wellbeing
- ❑ Pregnancy or early parenting
- ❑ Sleep improvement
- ❑ Stress reduction
- ❑ Substance use (e.g., Nicotine, drug, or alcohol recovery)
- ❑ Other (Specify): ______

For each group of features, please rank the importance of each feature for you when referring a patient to a mental health app. You can only use each number once within a group of features. Therefore, no two features should have the same rank.

App Engagement (Please read all choices within the section before making your selections. Use each rating number only once.)

	1 – Most Important	2	3	4	5	6 – Least Important
Fun, entertaining or interesting to use	○	○	○	○	○	○
Settings can be personalized (e.g., reminders, notifications, sound, content, challenges and goal setting, sharing options, etc.)	○	○	○	○	○	○
Content (e.g., visuals, language, design appeals to my personal preferences	○	○	○	○	○	○
Allows user input, provides feedback, and contains prompts	○	○	○	○	○	○
Allows user to be contacted by a mental health provider if needed	○	○	○	○	○	○
Provides availability of a coach	○	○	○	○	○	○

App Functionality (Please read all choices within the section before making your selections. Use each rating number only once.)

	1 – Most Important	2	3	4	5 – Least Important
Functions fast	○	○	○	○	○
Easy to learn how to use	○	○	○	○	○
Clear menu labels, icons, and instructions	○	○	○	○	○
Taps, swipes, pinches, scrolls (movement) that makes sense	○	○	○	○	○
Available in multiple languages	○	○	○	○	○

App Design (Please read all choices within the section before making your selections. Use each rating number only once.)

	1 – Most Important	2	3	4 – Least Important
Arrangement and size of buttons, icons, menus and content on the screen	○	○	○	○
Quality/resolution of the app graphics used for buttons, icons, menus, and content	○	○	○	○
Visually appealing	○	○	○	○
Narrator voice (e.g., gender, tone, accent, etc.)	○	○	○	○

App Information (Please read all choices within the section before making your selections. Use each rating number only once.)

	1 – Most Important	2	3	4	5 – Least Important
Content is well written and is relevant to the stated goals/topics	○	○	○	○	○
Quantity of the information is comprehensive but concise	○	○	○	○	○
Duration of the app sessions	○	○	○	○	○
Visual information (e.g., charts, graphs, images, videos) used to explain concepts is clear and logical	○	○	○	○	○
Information within comes from a legitimate or credible source	○	○	○	○	○

If there are other features you consider that are not listed in the tables above, please write them in here.

Using a few sentences, please describe a typical patient for whom you prescribe mental health apps. Consider the type and severity of the condition for which the app is ordered, how you introduce and integrate the app use into an overall treatment plan, and what factors you believe would lead to successful use by the patient.

Considering the patient you described above, which of the of the following do you discuss with the patient when referring a mental health app? (Check all that apply).

- ❑ How to download the app.
- ❑ When to download the app (e.g., during the visit, immediately after the visit, another time, etc.).
- ❑ How to navigate around in the app.
- ❑ The different types of app content.
- ❑ Best time to use the app.
- ❑ How to tailor or use the specific app content to meet their needs.
- ❑ How to use the app to set goals.
- ❑ Other (Specify) ______
- ❑ None of the above.

For the typical patient for whom you refer or order apps for mental health apps, what are the most challenging barriers to overcome for successful use?

Based on your experience, provide three key tips that you would give to a peer provider to facilitate successful use of mental health apps for their patients.

What is your current age in years?

______ (Years)

Gender

○ Female
○ Male
○ Other
○ Prefer not to say

Which of the following best describes your highest level of training?

○ Licensed clinical social worker
○ Licensed professional counselor
○ Licensed psychologist
○ Physician
○ Psychiatric nurse specialist
○ None of the above

What is your primary specialty?

○ Behavioral health
○ Primary care
○ Specialty care
○ None of the above

Since completing your clinical training, how many years have you been providing care?

________ (Years)

What age range of patients do you primarily serve? (Check all that apply.)

❑ ≤18 years
❑ ≥19 to < 45 years
❑ ≥45 to < 65 years
❑ ≥65 years

Which of the following mental health apps have you personally tried? (Check all that apply.)

❑ Calm
❑ Headspace
❑ myStrength
❑ Whil
❑ SilverCloud
❑ Thrive
❑ Don't know
❑ None of the above
.

Thank you for completing the survey! Please remember to submit your responses in order to receive your incentive.
